#  Effects of Pantoprazole on Systemic and Gastric Pro- and Anti-inflammatory Cytokines in Critically Ill Patients 

**Published:** 2012

**Authors:** Hamed Tabeefar, Mohammad Taghi Beigmohammadi, Mohammad Reza Javadi, Mohammad Abdollahi, Ata Mahmoodpoor, Arezoo Ahmadi, Hooshyar Honarmand, Atabak Najafi, Mojtaba Mojtahedzadeh

**Affiliations:** a *Department of Pharmacotherapy, Faculty of Pharmacy, Tehran University of Medical Sciences, Tehran, Iran. *; b *Anesthesiology and Intensive Care Department, School of Medicine, Tehran University of Medical Sciences, Tehran, Iran. *; c *Pharmaceutical Sciences Research Center, Faculty of Pharmacy, Tehran University of Medical Sciences, Tehran, Iran.*; d *Anesthesiology and intensive Care Department, School of Medicine, Tabriz University of Medical Sciences, Tabriz, Iran. *

**Keywords:** Pantoprazole, Cytokines, Stress-related mucosal damage prophylaxis, Critically ill patients

## Abstract

Stress-related mucosal damage (SRMD) is a significant cause of morbidity and mortality in critically ill patients due to the gastrointestinal blood loss. Prophylaxis of SRMD with proton pump inhibitors or histamine-2 blockers has gained widespread use in intensive care units. Both demonstrated to be effective in reducing clinically significant bleedings, while PPIs has shown to exert some anti inflammatory effects including the inhibition of producing pro-inflammatory cytokines. As cytokines have role in developing SRMD, the aim of this study was to evaluate the effect of PPIs on the inhibition of cytokine release following the critical illness.

A total of 27 critically ill patients with risk factors of developing stress ulcer and intragastric pH < 3.0 enrolled to this Randomized clinical trial study. Patients were randomly assigned in three treatment groups; group one received 40 mg of intravenous pantoprazole every 12 h for 48 h (four doses), group two received 80 mg of intravenous pantoprazole every 24 h continuous infusion for 48 h and the third group received 150 mg of ranitidine intravenously as 24 h continuous infusion for 48 h. Plasma and gastric juice samples were obtained at 0th, 12th, 24th and 48th h for the measurement of EGF, IL-1*β*, IL-6, IL-10 and TNF-*α*.

Pantoprazole infusion have decreased the plasma IL-1*β *concentrations (p = 0.041).

No other significant differences in concentrations of EGF, IL-6, IL-10 and TNF-*α *were detected.

There were reverse correlations between the intragastric pH with gastric juice IL-1*β *and TNF-*α *concentrations and a direct correlation between the intragastric pH and gastric juice EGF in pantoprazole groups.

Our data suggest that pantoprazole may have some anti-inflammatory effects on patients. However, the exact impact of this effect on patients should be assessed by further studies.

## Introduction

Stress-related mucosal disease (SRMD) is known to be a significant cause of morbidity and mortality in critically ill patients in the intensive care unit (ICU). Stress-related mucosal damage causes mucosal erosions and superficial hemorrhages in these patients or in those who are under extreme physiological stress, resulting mild to severe gastrointestinal blood loss.

Upper GI bleeding related to SRMD, estimated to affect 15% of patients in an ICU (1). Morbidity of SRMD and associated stress-related bleeding showed to double the length of stay in the ICU from 4 to 8 days (2). In critically ill patients who develops stress-related mucosal bleeding during the hospitalization, mortality rate varies in the range of 50-77%, which shows as much as 4 times higher than it is in ICU patients without this complication (3).

Splanchnic hypoperfusion is a major factor in the development of SRMD, which is resulted from a number of reactions produced by the body in response to the stress of critical illness, including sympathetic nervous system activation, increased catecholamine release and vasoconstriction, hypovolemia, decreased cardiac output, and release of pro-inflammatory cytokines (4, 5).

Stress of critical illness can induce the release of some inflammatory or anti-inflammatory cytokines and mediators (4, 6-9). Cytokines have role on development of SRMD through producing splanchnic hypoperfusion in critically ill patients (4, 5, 10). For example, mechanical ventilation can adversely affect GI trough the release of cytokines (5). In addition, plasma concentration of cytokines such as interleukin IL-6 may have a predictive value on patient complications or prognosis (11).

Efforts have directed at defining optimal therapy for stress ulcer prophylaxis in high-risk ICU patients (12-16). Establishing the adequate visceral perfusion and acid suppression therapy are the major preventive strategies. Acid suppression therapy with histamine-2 receptor antagonists (H2RAs) or proton pump inhibitors (PPIs) has shown to significantly decrease the occurrence of overt bleeding compared with placebo (17-19).

Proton pump inhibitors are at least as effective as H2RAs, and according to some of the previous trails, PPIs may be more effective in achieving the target intragastric pH more than 3.5 and preventing stress-related mucosal bleeding (4). In addition, PPIs have been found to have beneficial effects that cannot be explained by an increase in intragastric pH (20, 21). Proton pump inhibitors have been found to have anti-oxidant properties and direct effects on neutrophils, monocytes, endothelial, and epithelial cells that might prevent the inflammation (22-24). They are shown to have anti-inflammatory effects in ischemia/reperfusion (a major factor in development of SRMD) small intestinal injury unrelated to acid secretion (21). PPIs can modify the inflammatory reactions in helicobacter pylori infected patients (25).

Such effects were not obtained in comparative parallel studies with H2RAs (26). A number of mechanisms whereby PPIs can exert the anti-inflammatory effects unrelated to the inhibition of gastric acid production have been explicated in recent studies (20, 27). They may exert the anti-inflammatory effects by inhibiting the production of pro-inflammatory cytokines which provoke the inflammatory cells migration to diseased tissues (28). As cytokine release has role in the development of SRMD along with the acid secretion (4, 5), in this study for the first time, we assessed the SRMD acid suppression prophylactic therapy in aspect of probable effects of PPIs on cytokine release. The result of *in-vitro *studies have shown the effects of PPIs on decreasing the level of Interleukin (IL)-1*β*. The IL-6 and Tumor necrosis factor-*α *(TNF-*α*) (23, 29, 30) and some other studies have demonstrated the anti-inflammatory and gastric protective roles of IL-10 and Epidermal growth factor (EGF) (31, 32). In the current assay, we tried to find if there is any impact of PPIs on the release of these cytokines compared with H2RAs among the critically ill patients.

## Experimental

This study was a randomized clinical trial and was done at Imam Khomeini teaching hospital, affiliated to Tehran University of Medical sciences (TUMS) from April 2010 to August 2011. Patients who were ICU-admitted and required mechanical ventilation were recruited.

This trial is registered in www.anzctr.org.au, with the number of ACTRN12611000647932. The study protocol was approved by our institutional ethics committee (TUMS Pharmaceutical Sciences Research Center) and the written consent form was obtained from each patient’s closest family member. Twenty seven Patients were randomly assigned to 3 study groups according to a computer-generated table of random numbers. Group one received intravenous bolus pantoprazole 40 mg every 12 h for 48 h (four doses). Group two received 80 mg/day pantoprazole as continuous infusion for 48 h and the third group received 150 mg ranitidine as 24 h continuous infusion for 48 h.

Inclusion criteria were as follows: Non per oral (NPO) patients, with the need of mechanical ventilation, the presence of a nasogastric tube with a gastric position confirmed on the abdominal radiography and the baseline gastric juice with pH equal to or lower than 3.0 and the presence of at least one risk factor other than ventilation for a gastroduodenal stress ulcer that would commonly indicate the SRMD prophylaxis (*i.e*. shock, severe sepsis, burns, head trauma, coagulopathy, major surgery) (33, 34). The patients did not receive any H2-blocker, proton pump inhibitor, or antacids for the last two days and the enteral feeding was not allowed during the study period. Patients younger than 18 years and patients with renal or hepatic failure were excluded from the study.

All patients were included in trial within the first 12 h after the admission to the ICU. At 0, 12, 24 and 48 h after the administration of the mentioned drugs, 10 mL of gastric juice was aspirated by 50 mL of syringe through the nasogastric tube and dropped out to make sure that the aspirate is clear from nasogastric tube contents. A second dose of 10 mL aspiration was obtained as the sample for cytokines measurements. The pH of gastric juice was determined by pH-indicator strips (MERCK KgaA, Germany). Five mL of blood specimens for the measurements of cytokines were also collected. Patient’s daily hemodynamic and laboratory data were recorded for calculating the sequential organ failure assessment (SOFA) during the study.

*Cytokine measurement*

Tumor necrosis factor-*α*, IL-1*β*, IL-6, IL-10 and EGF were determined in duplicate, by enzyme immunoassay method (Boster immunoleader, Wuhan Boster Biological Technology, China). Blood specimens were collected into plastic tubes with EDTA and plasma was separated by centrifugation at 10,000 × g for 10 min and at 4°C. Gastric juice particles were separated by centrifugation at 4,000 × g for 3 min. All gastric juice and plasma samples were stored at -75°C before the analysis. The limits of assay detection were 15.6 pg/mL for TNF-*α*, 1.56 pg/mL for IL-1*β*, 4.69 pg/mL for IL-6, 3.4 pg/mL (plasma) and 7.8 pg/mL (gastric juice) for IL-10 and 4.7 pg/mL for EGF.

*Statistical analysis*

Characteristics of patients are represented as mean ± SD. Statistical analyses of data between the characteristics of patients were performed by one-way analysis of variance. A repeated measures Analysis of Variance (ANOVA) model was used to analyze the data (SPSS 17.0, Chicago, IL, USA) to compare mean values between the groups and time. Pearson’s correlations were used to assess any associations between variables concerning cytokines levels and pH within the group of study. A p-value less than 0.05 was considered significant. 

In Pantoprazole infusion group with increases in intragastric pH, plasma concentrations of TNF-*α *were decreased (A and B). In Pantoprazole bolus and infusion groups with increases in intragastric pH, gastric juice concentrations of TNF-*α *were decreased (C, D and E). Concentrations of EGF were increased with increases in intragastric pH in Pantoprazole bolus group.

## Results

*Patient characteristics*

Group 1 included 11 patients and groups 2 and 3 both included 8 patients. Patient characteristics at the admission are shown in Table 1. No statistical differences in age, sex and basal pH and SOFA were found between the treatment groups. All patients were incubated and had respiratory failure as the most important risk factor for SRMD. Risk factor distributions are shown in Table 2.

**Table 1:** Patient characteristics at the time of admission.

**Characteristics**	**Ranitidine Infusion (n = 8)**	**Pantoprazole Bolous (n = 11)**	**Pantoprazole Infusion (n = 8)**	**p-value**
Age-year	50.4 ± 8.0	47.0 ± 11.0	39.7 ± 8.0	0.17 (NS)
Sex (Male/Female)	6/2	9/2	6/2	0.92 (NS)
pH0	2.25 ± 0.56	2.53 ± 41	2.24 ± 0.75	0.47 (NS)
SOFA0	7.00 ± 1.69	5.36 ± 1.91	6.75 ± 1.98	0.13 (NS)

NS: Not Significant; pH0: pH at base line; SOFA0: sequential organ failure assessment at baseline.

**Table 2:** Risk factors for clinically important bleeding

**Risk factor**	**Ranitidine (n = 8)**	**Pantoprazole Bolus (n = 11)**	**Pantoprazole Infusion (n = 8)**
Coagulopathy	0	0	0
Shock	0	3	1
Sever Sepsis	1	0	1
Trauma	3	4	6
Major Surgery	5	6	5
Respiratory Failure	8	11	8

The risk factor distribution was similar in the three study groups, except that more patients with shock and trauma assigned to receive pantoprazole bolus or infusion respectively.

*pH measurement*

**Figure 1:**
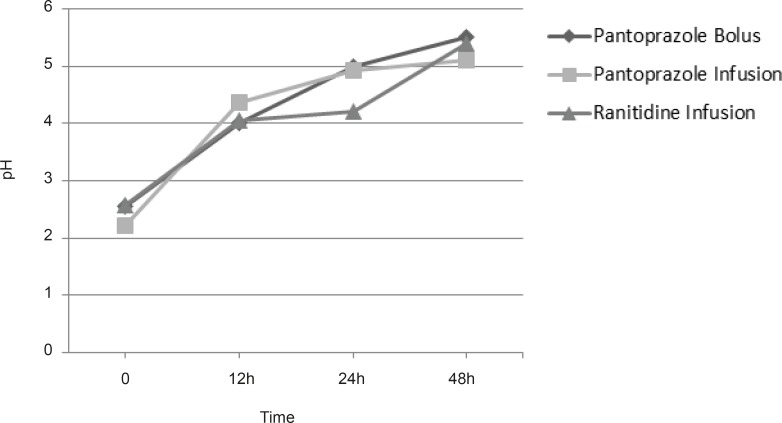
Mean intragastric pH in groups, p > 0.05.

All patients enrolled to the study had intragastric pH less than 3.0 at the time of admission. There were no statistically significant differences in intragastric pH between the groups at the admission time. We did not find any significant differences in pH between groups in times 0, 12, 24 and 48 h. Figure 1 shows mean intragastric pH in groups.

*Assessment of the effects of drugs administrations on plasma and gastric juice cytokines*

**Figure 2:**
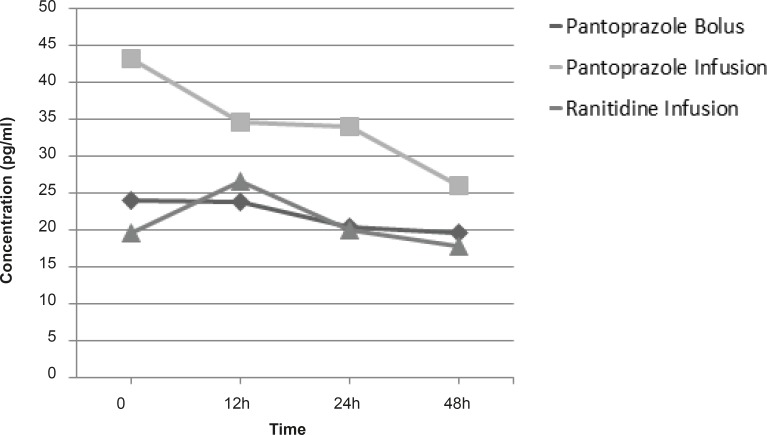
Mean IL-1*β *concentrations in groups (p < 0.05).

In analysis of the effect of drugs administrations on plasma and gastric juice concentrations of TNF-*α*, EGF, IL-1*β*, IL-6 and IL-10, group of study only had a significant effect in decreasing plasma concentrations of IL-1*β *and the patients who had received pantoprazole continuous infusion had significantly decreased IL-1*β *concentrations rather than other groups(p = 0.041) (Figure 2). For other cytokines (TNF-*α*, EGF, IL-6 and IL-10) we did not find any significant difference in plasma and gastric juice between the groups during the study.

*Correlation between intragastric pH and cytokines in plasma and gastric juice within each group*

**Figure 3:**
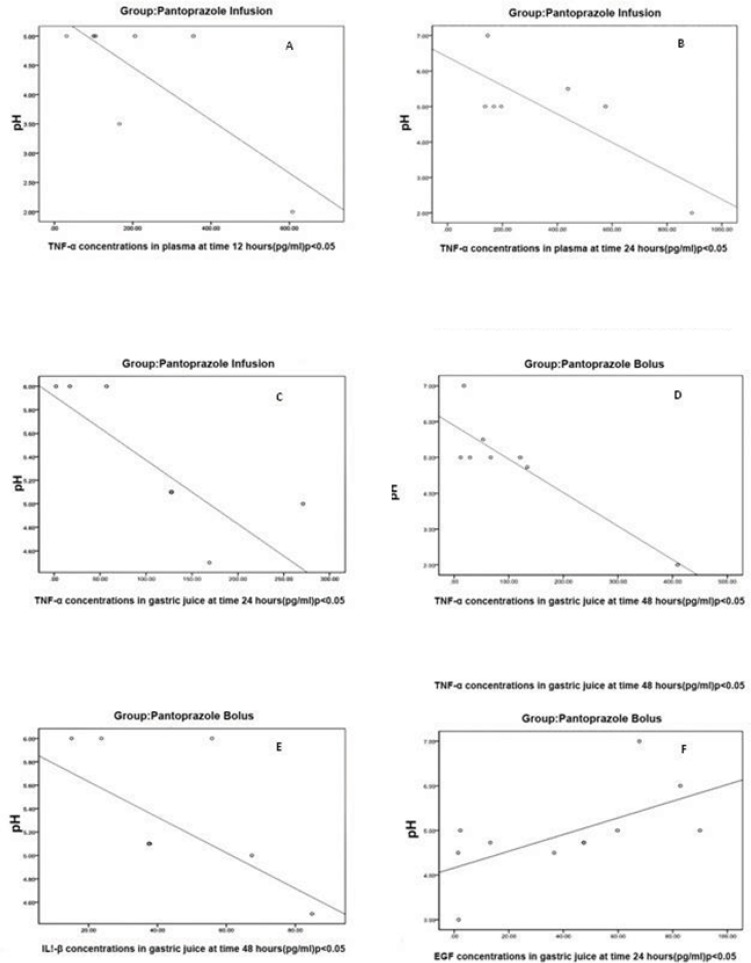
Correlations between intragastric pH and cytokines concentrations within each study group.

In assessment of intragastric pH correlation with plasma and gastric juice cytokines within each study group, in groups who had received pantoprazole (groups 1 and 2) we found significant correlations between pH and cytokines in some time points (Figure 3). In pantoprazole infusion group, we found reverse correlations between pH and plasma TNF-*α *concentrations at 12 and 24 h (*r *= -0.76, p < 0.048 and *r *= -0.78, p < 0.041 respectively).

In pantoprazole bolus group at 48 h, there was a reverse correlation between intragastric pH and gastric juice concentration of IL-1*β *(*r *= -0.60, p < 0.049). In addition, a reverse correlation between the intragastric pH and gastric juice concentrations of TNF-α at 24 and 48 h were found in pantoprazole infusion and pantoprazole bolus groups respectively (*r *= -0.89, p < 0.003 and *r *= 0.80*, *p < 0.003). Finally, for EGF, there was a direct correlation between EGF and gastric juice pH in pantoprazole group at 24 h, (*r *= 0.6, p < 0.04).

## Discussion

Our study showed that continuous infusion of pantoprazole could decrease the IL-1*β *plasma concentrations significantly, while this effect was not observed in ranitidine continuous infusion or pantoprazole bolus treatment groups. Continuous infusion of pantoprazole could significantly decrease the plasma concentration of IL-1*β *as compared with bolus, which may show the probable value of the administration method and persistence of acid suppression on cytokines outflow during the study period. In assessment, the correlations between the intragastric pH, plasma and gastric juice concentrations of cytokines within each group, in patients who received pantoprazole (groups 1 and 2), is indicated that patients with higher intragastric pH had lower inflammatory cytokine outflow (Figure 3). We did not find similar correlations with ranitidine. This might be an indicator of possible pantoprazole anti-inflammatory effect.

Interleukine-1*β *and TNF-*α *are pro-inflammatory cytokines, both are being produced at the sites of local inflammation. They are inducers of endothelial adhesion molecules, which are essential for the adhesion of leukocytes to the endothelial surface of diseased tissues, initiating the cascade of inflammatory mediators (34). Proton-pump inhibitors can affect the transmigration of leukocytes from vessels to the inflammatory sites (27). By this mechanism, they may prevent inflammatory cell migration to gastric cells and consequent decrease in cytokine release in that site. Epidermal growth factor is a potent cytoprotective agent, an important element of ulcer healing. Under the gastric inflammation, gastric mucosal cell turnover such as apoptosis or proliferation is frequently regulated by local growth factors like EGF.

The elevations of EGF concentration along with a rise in intragastric pH with pantoprazole could further prove the probable anti-inflammatory effects of this agent. These beneficial effects could be indicative of pantoprazole anti-inflammatory effects in critically ill patients. However, in many experimental studies which have been conducted regarding the anti-inflammatory effects of PPIs, higher concentrations of pantoprazole were used that could not be obtained in human body with normal dosages of this agent. As the patients who are hospitalized in ICU suffer from multiple critical conditions and are individually being treated with different doses of medications which strongly depends on each patient’s exclusive physiological and hemodynamic conditions (even different treatments for patients with the same disease), controlling the variables such as the patient’s illness severity and medications types and drug dosages make the controlling of such a study conditions ultimately difficult and even impossible. Therefore, we tried to specify these on the basis of primary assessment of patient’s illness severity by means of “SOFA”, primary pH level and SRMD prophylaxis indications (Tables 1 and 2). It could be assumed that conducting this study with one group of patient candidate for SRMD prophylaxis (*e.g. *just traumatic patients or patients who have undergone major surgeries) could help unify the study set up with respect to the kind of disease and medications. Thereupon, broader clinical studies in this regard necessitated in future to clarify this effect.
